# Magnetic phase transition from paramagnetic in Nb_2_AlC-MAX to superconductivity-like diamagnetic in Nb_2_C-MXene: an experimental and computational analysis

**DOI:** 10.1039/d0ra04568c

**Published:** 2020-07-07

**Authors:** Zaheer Ud Din Babar, Jameela Fatheema, Nimrah Arif, M. S. Anwar, Sundus Gul, Mudassir Iqbal, Syed Rizwan

**Affiliations:** Physics Characterization and Simulations Lab (PCSL), Department of Physics, School of Natural Sciences (SNS), National University of Sciences and Technology (NUST) Islamabad 44000 Pakistan syedrizwan@sns.nust.edu.pk syedrizwanh83@gmail.com +925190855599; Department of Chemistry, School of Natural Sciences (SNS), National University of Sciences and Technology (NUST) Islamabad 44000 Pakistan; Department of Materials Science and Metallurgy, University of Cambridge CB3 0FS Cambridge UK

## Abstract

Transition metal carbides (TMCs) have recently emerged as competent members among the family of two-dimensional (2D) materials, owing to their promising applications. There are many promising applications of MXenes; however, their magnetic properties lack a wide margin, both experimentally as well as theoretically, which needs to be investigated for potential use in spintronics. In this study, we carried out a comprehensive etching process *via* selective extraction of Al layers from Nb_2_AlC-MAX using a wet electrochemical route under well-optimized conditions to obtain fine 2D-Nb_2_C MXene sheets. Structural analysis using X-ray diffraction (XRD) confirms the effective removal of Al followed by confirmation of a 2D layered structure from morphological analysis using scanning electron microscopy (SEM). Zero-field-cooled (ZFC) and field-cooled (FC) measurements of MAX and MXene at different field strengths were performed using a superconducting quantum interference device (SQUID). Magnetic measurements reveal the paramagnetic nature of Nb_2_AlC-MAX measured under 5 mT; however, this changes to a clear superconductor-like diamagnetic behavior with a shift of the magnetization from positive to negative values at low temperatures when measured under 5 mT and 10 mT for Nb_2_C MXene. The diamagnetism, however, is changed to paramagnetism at 100 mT, which shows the existence of critical fields known typically for a type-II superconductor. To gain an insight into this unusual behavior in MXene, density functional theory (DFT) first-principles calculation was also performed in Wein2K software using spin-polarized generalized gradient approximation (sp-GGA). The magnetic moment of the compound is calculated to be negative, which corresponds well with the experimental finding and suggests that the negative magnetic moment originated from the d-orbital of Nb_2_C. The present report provides a pathway to deeply understanding the existence of superconductivity-like diamagnetic behavior in Nb_2_C MXene, which is useful for future magnetic applications.

## Abstract 2

Transition metal carbides (TMCs) have recently emerged as competent members among the family of two-dimensional (2D) materials, owing to their promising applications.

## Graphical abstract

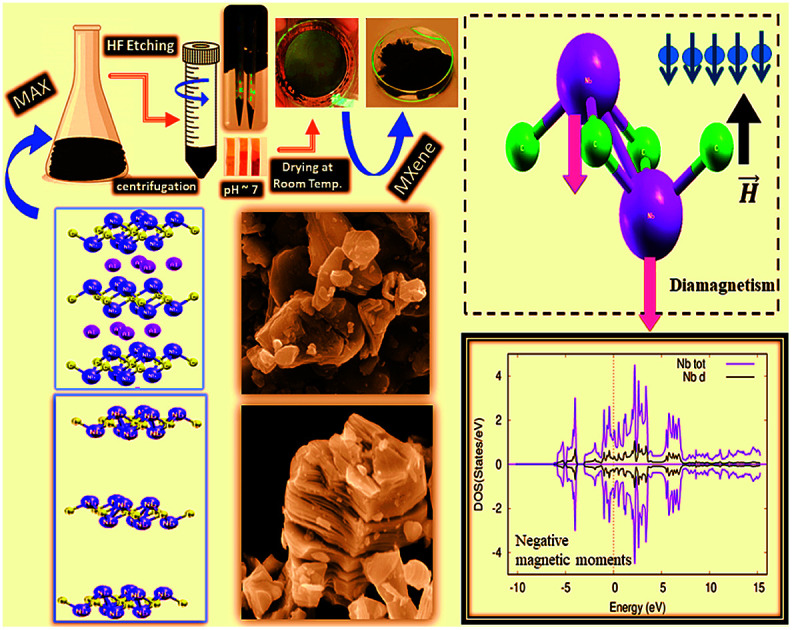

In two-dimensional (2D) materials, magnetism at the nanoscale is at the forefront of many cutting-edge technological applications, such as spintronic devices. Naguib *et al.* have synthesized a new class of two-dimensional materials, known as MXenes (M_*n*_X_*n*+1_), discovered in 2011, giving a possibility of magnetism in such 2D materials and their promising uses in spintronic devices.^1^ These 2D layer structured early transition metal carbides and/or nitrides are known as MXenes, so named to indicate their structural similarities with graphene.^2^ MXenes are derived from 3D MAX phases (space group *P*6_3_/*mmc*) in which “M” is an early transition metal, *e.g.*, Ti, Ta, V, *etc.*; “A” is mainly a group 13 or group 14 element (group III-A or IV-A), *e.g.*, Si, Al; “X” is a carbide, nitride or can be both; and *n* = 1, 2, 3 represents the number of layers, forming 211, 312 and 413 phases.^3–5^ Over the past decade, this new material has gained enormous attention, thus developing an entirely new research field to disclose the properties of the 2D state of this material. The materials in the 2D regime own a cluster of astonishing physical properties as compared to the 3D nature, but intrinsic two-dimensional magnetism has proved to be challenging. As 2D semiconductors have revamped the field of electronics, similarly, magnetism in 2D materials could remodel spintronic devices that can employ a spin degree of freedom.^6,7^

Nb_2_C MXene was first synthesized by Naguib *et al.*, but they just reported its electrochemical activity as a promising electrode material.^4^ Further work has been reported in Nb_2_C with various biomedical applications, energy storage, supercapacitors, and nanoelectronics.^9–19^ As far as the magnetism in such 2D MXenes is concerned, it remains less investigated, and this research void needs to be filled. Recently, Babar *et al.* reported the observation of superconductivity in as-prepared powdered Nb_2_C for the first time, with the highest onset transition temperature *T*_c,onset_ = 12.5 K among the MXene family. However, the authors did not discuss the magnetic nature of the parent Nb_2_AlC MAX itself and did not reason for the presence of unusual magnetic effects in MXene.^8^ MXenes are favorable members of 2D magnetism, and different magnetic natures are computationally predicted in various carbide and nitride MXenes.^7,20^ The existence of novel room-temperature ferromagnetic order in doped MXene and the coexistence of different magnetic phases in MXene, along with experimental evidence, indicate its potential of hosting diverse magnetic natures.^21,22^ Considerable research has been focused on these 2D structures due to their importance and favorable applications, such as spintronics. MXene could provide a vast platform for exploring the magnetic properties and is one of the best candidates that can host superconductivity as compared to other members of the 2D family. Experimental studies are generally dependent upon numerous variables, thus affecting the research pace. However, density functional theory-based first principles calculation and theoretical simulations are a successful way to examine and foresee the properties of low-dimensional materials. This provoked us to theoretically explore superconductivity in Nb_2_C and their validation through superconductivity measurements of experimentally synthesized Nb_2_C MXene. In this work, we report a systematic etching mechanism of Nb_2_C MXene to obtain fine 2D sheets *via* a chemical etching route using hydrofluoric (HF) acid. Structural and morphological studies using the X-ray diffraction technique (XRD), scanning electron microscopy (SEM) and elemental analysis by energy-dispersive X-ray spectroscopy (EDX) show the effective removal of Al from the parent 3D-Nb_2_AlC MAX, thus revealing an accordion-like sheet structure. Optical analysis indicates a significant reduction in bandgap after chemical etching. Magnetic properties were carried out to observe the signatures of superconductivity (a perfect diamagnetic state, negative magnetic moment) and its magnetic nature at room temperature. To study the magnetic nature of as-prepared powder-form Nb_2_C, density functional theory (DFT) first principles calculation was carried out through Wein2K using spin-polarized generalized gradient approximation (sp-GGA). The magnetic moment of the compound is calculated to be −0.00485, which although but small is important, as the value is negative, which is an indication of the presence of diamagnetism in Nb_2_C. Here, the detailed chemical etching process, magnetic properties of Nb_2_AlC MAX and its effect on magnetic phase of Nb_2_C MXene, and the density functional theory calculation are reported, which were not discussed by Babar *et al.* in ref. 8.

## Results and discussion

The structural and phase purity of resultant MXene powder was characterized by X-ray diffractometer (XRD, Bruker, D8 Advance, Germany) using Cu-Kα radiation. X-ray diffraction (XRD) patterns of Nb_2_AlC MAX and 2D layered MXene are shown (see Fig. 2a and b). There are two main peaks in the XRD pattern of MAX, the first peak at *θ* = 12.9° and the second peak at *θ* = 38.8°. After chemical treatment, the total disappearance of the second peak indicates the removal of Al from the parent compound.

The broadening of the peak at *θ* = 12.9° and its downshifting indicates that MAX is converted into MXene. Middle Al layers can be selectively removed due to the weaker nature of Nb–Al bonds than the Nb–C bond in the Nb_2_AlC MAX phase after high-temperature treatment with hydrofluoric acid.^4,23^ Weaker hydrogen bonding in M–Al layers comes in place of stronger metallic bonds that result in facile separation of sheets upon HF treatment and subsequent intercalation of water molecules during the washing process. These different relative bond strengths allow the selective removal of Al without affecting the M–C layer,^8,23,24^ resulting in hexagonally stacked 2D sheets of Nb_2_C MXene. The elemental composition, obtained from energy-dispersive X-ray spectrum (EDX), is shown in the table provided in Fig. 2. The EDX elemental mapping shows a significant reduction of Al content after HF treatment, showing the successful etching process. Both O and F are also present in MXene as functional groups, which are unavoidable during the synthesis process. It is worth noting that aluminum in Nb_2_AlC is very active and could easily adsorb oxygen. After the etching, Al is dissolved, and oxygen contents associated with Al are therefore decreased. The high content of O in MAX phases may be attributed to several reasons. Oxygen contaminations in primary precursors (*i.e.*, metal powders) emanate oxygen impurity phases such as alumina.^25^ Experimental studies on similar MAX phases reveal that oxygen could possibly substitute carbon without affecting the MAX structure.^26^ J. Rosen *et al.* have reported oxygen as a potential element in comparison to nitrides or carbides that could dictate the carbide/nitride lattice sites.^27^ T. Liao *et al.* have also indicated that the treatment of MAX at high-temperature conditions could trigger oxygen to reside at c-sites.^28^ They reported 50% of carbon being replaced by oxygen content. Moreover, due to the high affinity of aluminum towards oxygen, weighing the initial precursors in an open environment or MAX precursors may lead to the formation of Al_2_O_3_ even if the synthesis is carried out in vacuum.^28,29^ The results also suggest that the formation of such impurities unintentionally incorporate significant oxygen content. Elevated temperature treatment of MAX phase with strong HF etchant leads to the removal of Al and its associated content, resulting in a more ordered structure.^30^

**Fig. 1:**
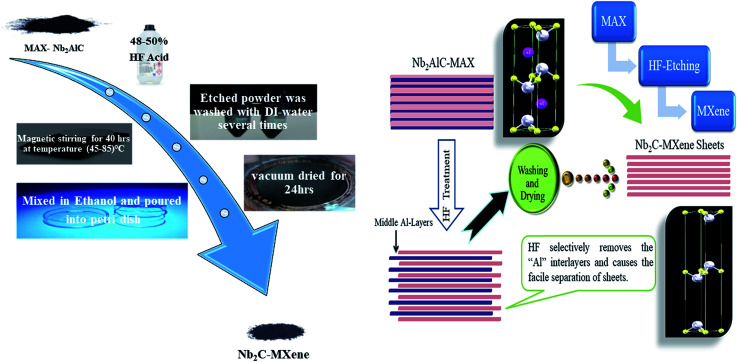
Detailed synthesis schematics and etching mechanism to obtain 2D Nb_2_C-MXene sheets.

**Fig. 2:**
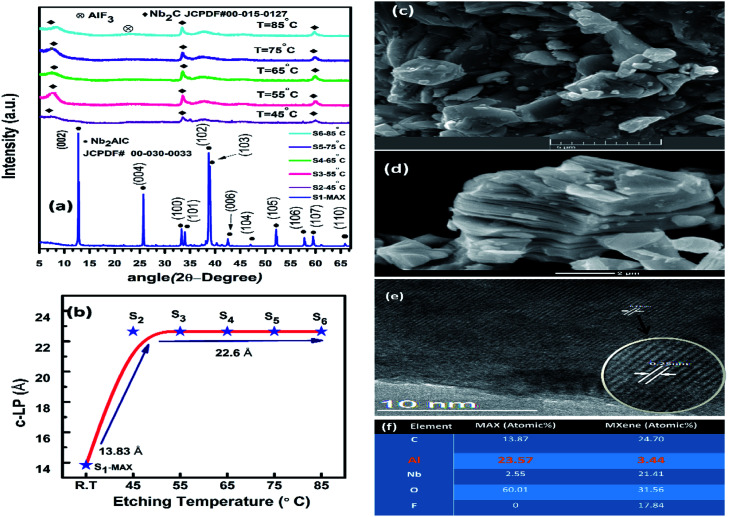
(a) XRD pattern of S_1_ (pure MAX), S_2_ (MXene etched at 45 °C), S_3_ (55 °C), S_4_ (65 °C), S_5_ (75 °C), S_6_ (85 °C). (b) Variation of *c*-LP of MAX and MXene for different sample series. (c and d) SEM images of MAX and MXene. (e) TEM image of Nb_2_C MXene flakes showing typical layered structure. (f) Table showing the atomic percentage of elemental composition of MAX and MXene obtained from EDX.

To observe the morphology of the sheets, scanning electron microscope (SEM) images at different resolutions were recorded using a field emission electron microscope (FESEM, VEGA3-TSCAN) operated at 20 kV. Structural morphological images, determined by scanning electron microscope (SEM) of MAX and MXene, are shown in Fig. 2c and d, respectively, showing a layered structure of the resultant MXene compound having a typical MXene morphology.^31–35^ Transmission electron microscopy (TEM) images of Nb_2_C-MXene are shown in Fig. 2e, clearly revealing the typical MXene layered structure. The exfoliated 2D-Nb_2_C MXene sheets are transparent and quite thin, as observed by TEM imaging. The variation of *c*-LP of MAX to MXene is presented in Fig. 1b as *c*-LP was increased to 22.6 Å from 13.83 Å (MAX phase), which satisfies the facile separation of the sheets after HF treatment.^4,36,37^

**Fig. 3:**
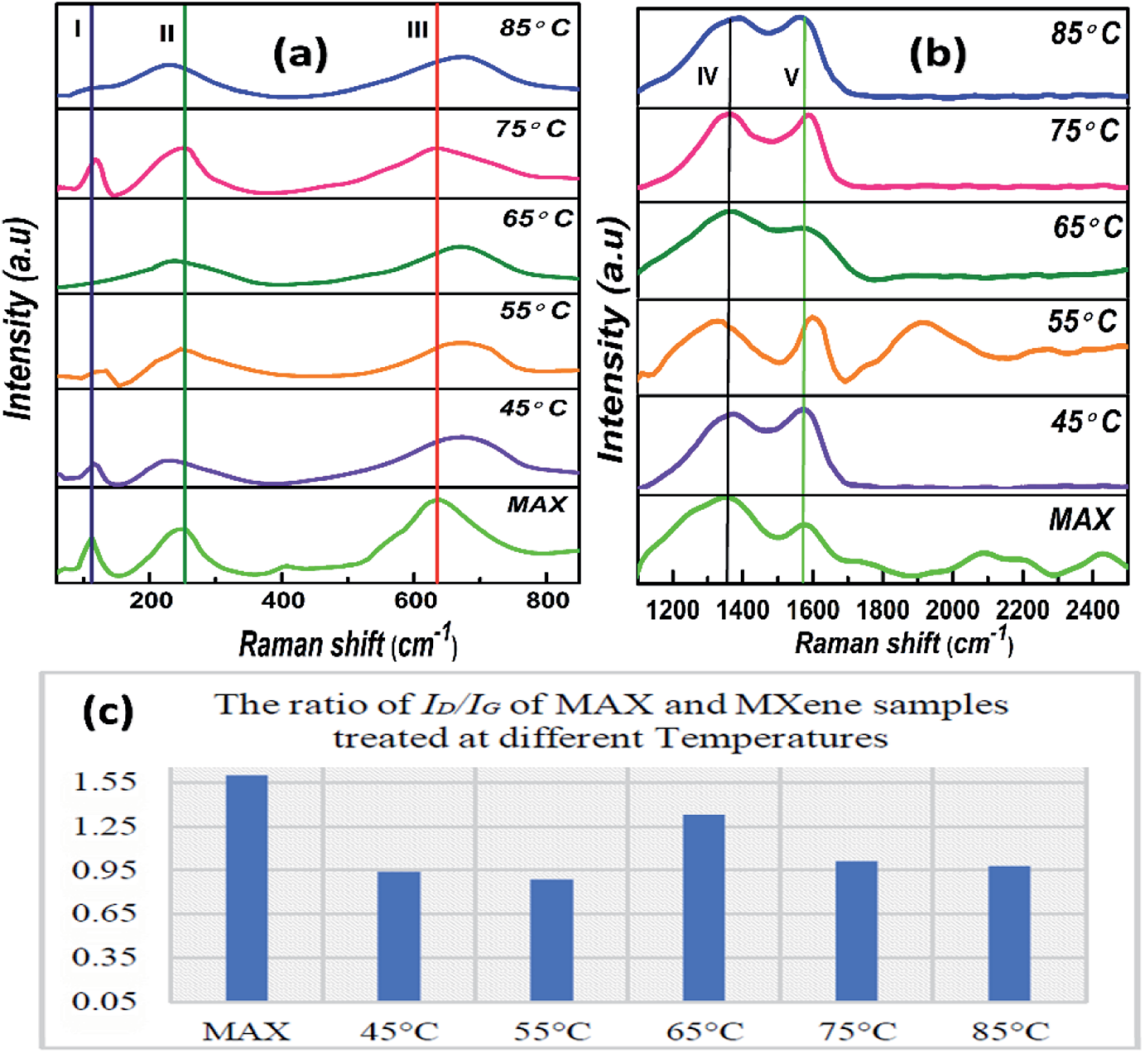
(a) Raman spectra of (S_1_) MAX and (S_2_) MXene at 45 °C, (S_3_) 55 °C, (S_4_) 65 °C, (S_5_) 75 °C, and (S_6_) 85 °C, (b) D and G-bands, (c) *I*_D_/*I*_G_ ratio of MAX and MXene at different temperatures.

Raman spectra were measured with Horiba Scientific, Xplora Raman analyzer with a laser wavelength of 532 nm in the region of 200–2500 cm^−1^. Fig. 3a and b shows the Raman of Nb_2_AlC before and after HF treatment done at various temperatures. The maximum number of peaks is downshifted, broadened, and shifted to higher wavenumber after terminating with F and OH groups, which indicates a strengthening of the bond between atoms.^38–41^ Peaks I and II in MAX are suppressed after HF treatment, indicating the removal of Al atoms or exchange of Al atoms by lighter atoms.^42^ Peak III was broadened and downshifted, which is related to C atoms.^43^ Peaks IV and V are representative of D and G bands of carbon species. The D band characterizes carbon disordered structure to sp^3^ hybrid carbon, and G is the graphitic band or sp^2^ carbon.^44^ The ratio of intensity of *I*_D_ to *I*_G_ tells us about the crystallinity defects, showing more ordered MXene phase than the MAX phase.^45,46^ The *I*_D_/*I*_G_ ratio of intensities in Fig. 3c tells us about the crystalline defects, which shows more ordered MXene phases than the MAX itself. This ratio is 1.6 for MAX, and it varies between 0.89 to 1.33 for MXene at different temperatures.^45^ This shows that the sample obtained and carried forward at 55 °C is more ordered, with less defect density as compared to the other samples synthesized at different temperatures.

**Fig. 4:**
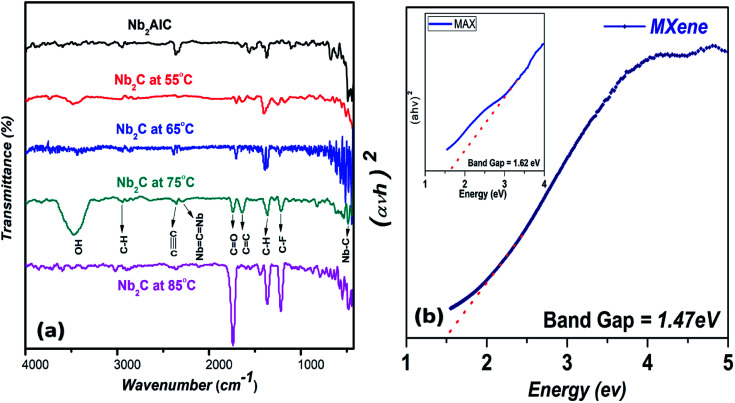
(a) Fourier transform infrared (FTIR) spectra of MAX and Nb_2_C-MXene synthesized at different conditions. (b) Band gap analysis of MAX and MXene.

The Fourier transform infrared (FTIR) spectra of Nb_2_C etched at different temperatures, *i.e.*, 55 °C, 65 °C, 75 °C and 85 °C, and are compared to the FTIR spectrum of pure MAX (Fig. 4a), indicating three absorption bands at 2357 cm^−1^, 2295 cm^−1^, and 483 cm^−1^, respectively, which are common and appear in all samples. These bonds can be attributed to the triply bonded carbons, Nb=C=Nb, and Nb–C, respectively.^47^ It is to be noted that the FTIR spectra of Nb_2_C is irrelevant to the etched temperature and shows a similar trend. A bond at 1643 cm^−1^ can be attributed to unsaturation mainly due to the C=C bond in the MXene structure. The presence of C–H bonds can also be indicated by the bands at 2947 cm^−1^ and 1363 cm^−1^. Based on the spectra and presence of different functional groups, surface functionalization can be observed in our MXene sheets due to the presence of a distinct C=O band at 1734 cm^−1^ and C–F band at 1211 cm^−1^.^48,49^ The absence of these bonds in the spectrum of the MAX phase shows that the etched MXene may have C=O, C–F, and O–H surface terminations.^50^

Tauc's plot was used to calculate the band gap of MAX and MXene using following equation:

1 *αhν* = *A*(*hν* − *E*_g_)^*n*^

where *h* = Planck's constant, *A* = constant, *ν* = the frequency of irradiated light, *E*_g_ = the bandgap energy, and *α* = the absorbance of light.^51^ The bandgaps of MAX and MXene were obtained by plotting (*αhν*)^2^ against bandgap energy *E*_g_.^52–56^ The bandgaps of MAX and MXene came out to be 1.62 eV and 1.47 eV, respectively, as shown in Fig. 4b. The reduction in the bandgap from MAX to MXene can be attributed to the presence of different surface terminations (with O, OH, and F), with bandgaps ranging from 0.45 eV to 1.8 eV.^4,57–60^

**Fig. 5:**
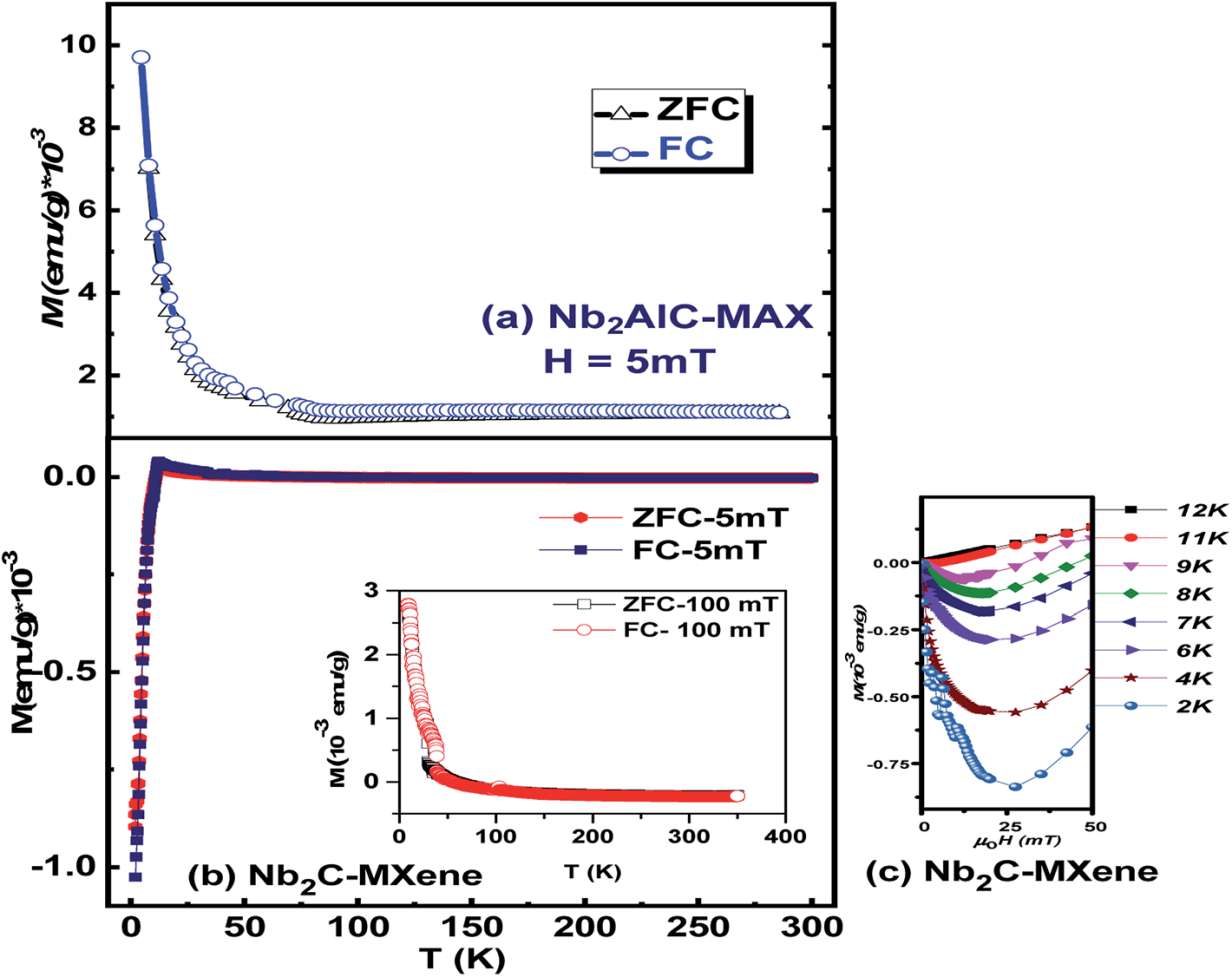
(a) ZFC–FC measurements of MAX at a field of 5 mT. (b) ZFC–FC measurements of MXene at 5 mT and 10 mT with inset of ZFC–FC curves at a field strength of 100 mT. (c) Variation of magnetic behavior with increase in temperature.

The magnetic nature of MXene has remained a less-explored research field, with only a few reports available on its magnetic nature. Their magnetic nature is predicted based on the magnetic transition metal elements (*e.g.*, Cr, Mn, V, Fe, and Ni) or in the form of any doped configurations. There exists a large gap between experimental studies and their theoretical verification, or *vice versa*. Due to limitations in synthesis techniques, there were no experimental reports on the magnetic properties of as-prepared MXene. Until recently, Babar *et al.* have synthesized Nb_2_C MXene and among the MXene family. In this report, the magnetic nature of Nb_2_AlC-MAX and Nb_2_C MXene is shown. Fig. 5a and b show the zero-field-cooled (ZFC) and field-cooled (FC) magnetization curves measured for Nb_2_AlC MAX and Nb_2_C MXene, respectively, under an applied magnetic field of 5 mT using a SQUID magnetometer (Quantum Design, MPMS). From these curves, one can see that the magnetic behaviors of MAX and MXene are different.

The FC–ZFC curves of MAX correspond to a paramagnetic material in which the magnetization increases positively with the decrease in temperature, whereas the FC–ZFC curves of MXene are opposite; the magnetization reverses its direction at a critical temperature and becomes negative at low temperatures. This trend of MXene fits well for a typical superconductivity-like diamagnetic response of a material. Recently, Babar *et al.* reported the Meissner effect in Nb_2_C MXene, which showed typical type-II superconductivity in the material with an onset transition temperature of 12.5 K. However, the authors did not discuss the origin of this behavior, as it might be inherent to the Nb_2_AlC MAX itself, and the contribution of the spin of the constituent elements needed to be discussed. Here, the magnetization *vs.* temperature (*M*–*T*) trend of MAX does not show any diamagnetic transition at any temperature, but a paramagnetic behavior.^61–63^ Hence, the MAX structure itself is not responsible for the previously observed superconductivity-like diamagnetism in MXene.

Contrary to the MAX phase, the FC–ZFC curves shown in Fig. 5b clearly indicate a magnetic phase transition showing a clear diamagnetic behavior that gives traces of the superconductivity effect in MXene. The inset of Fig. 5b indicates *M*–*T* curves of Nb_2_C MXene at a much higher applied field of 100 mT, at which the magnetization becomes positive at low temperature and is no longer a diamagnetic material. The sharp upward trend in magnetization, as well as the splitting between ZFC–FC curves at low temperature, indicates a significant paramagnetic contribution arising from the Nb ions.^64^

This also gives an indication of the existence of a threshold field that supports the presence of superconductivity in Nb_2_C MXene, as the threshold field corresponds to a type-II superconductor.^8^Fig. 5c shows the quadrant of magnetization *vs.* magnetic field (*M*–*H*) curves measured at 2 K, 4 K, 6 K, 7 K, 8 K, 9 K, 11 K and 12 K. The curves clearly show a transition of magnetization from low-negative to high-negative, and then to low-negative values, representing the presence of Meissner effect at these temperatures as well as the presence of the respective threshold fields. At 12 K, the *M*–*H* response is linear to the field and refers purely to a paramagnetic curve, indicating the transition temperature.

Several theoretical studies have been carried out to explore the magnetic properties of different MXenes. The Cr-based carbide and nitride MXenes are predicted to be magnetic according to theoretical studies carried out by M. Khazei *et al.*, and their electronic properties were reported to be altered upon adding the surface terminations.^65^ Different functionalized MXenes are predicted to be semiconductors with the bandgap range of 0.25–2 eV. Bare MXenes (MXenes without surface terminations) are supposed to have metallic nature, but the OH, O or F terminated MXenes show semiconductive nature, with bandgaps ranging from 0.05–1.8 eV.^66,67^ The asymmetric functionalized MXene, such as the Janus Mn_2_N, is a ferromagnet, while Mn_2_C is reported to be anti-ferromagnetic.^68^ Zhang *et al.* have reported the paramagnetic nature of as-prepared Ti_3_C_2_ and its magnetic variation on different synthesis conditions.^69^ Akgenc *et al.* theoretically pointed out the ferromagnetic half-metal and antiferromagnetic semiconductor nature of Ti_2_C MXene.^70^ Since the magnetic behavior of 2D nanostructures could be intrinsic or defect-induced, the wet chemical etching route could produce 2D sheets with intrinsic magnetic properties with high-yield and low-cost mass production.^71^ Considering the above findings, the magnetic behavior of our sample is found to be independent of the surface functionalization, providing the clue towards the intrinsic behavior of our Nb_2_C MXene. Its superconductivity-like behavior (the Meissner effect and the perfect diamagnetism at low temperature, negative magnetic moment) is calculated by density functional theory calculations.

Our group has recently reported a detailed experimental and theoretical analysis on stable ferromagnetism in Ti_3_C_2_ MXene. This report carries out experimental magnetic analysis with theoretical validations of doped and undoped Ti_3_C_2_ MXene.^72^ To further explore the reason for the existence of the observed superconducting-diamagnetism in Nb_2_C MXene (niobium carbide MXene), the structure was simulated in WIEN2k package, which employs the full-potential linear augmented plane wave method.^73,74^ The structure was optimized for the *c*-lattice parameter obtained from XRD analysis, where the space group is *P*6_3_/*mmc* and the positions for niobium (Nb) and carbon (C) are (1/3, 2/3, *u*) and (0, 0, 0), respectively. For the minimization of the structures, 500 *k*-points were used in the irreducible Brillouin zone, with a *k*-mesh of 14 × 14 × 2, while the self-consistent field was also performed at the same number of *k*-points. The function that is used for solving Kohn–Sham equations is spin-polarized Perdew–Burke–Ernzerhof generalized gradient approximation (PBE-GGA).^75^

**Fig. 6:**
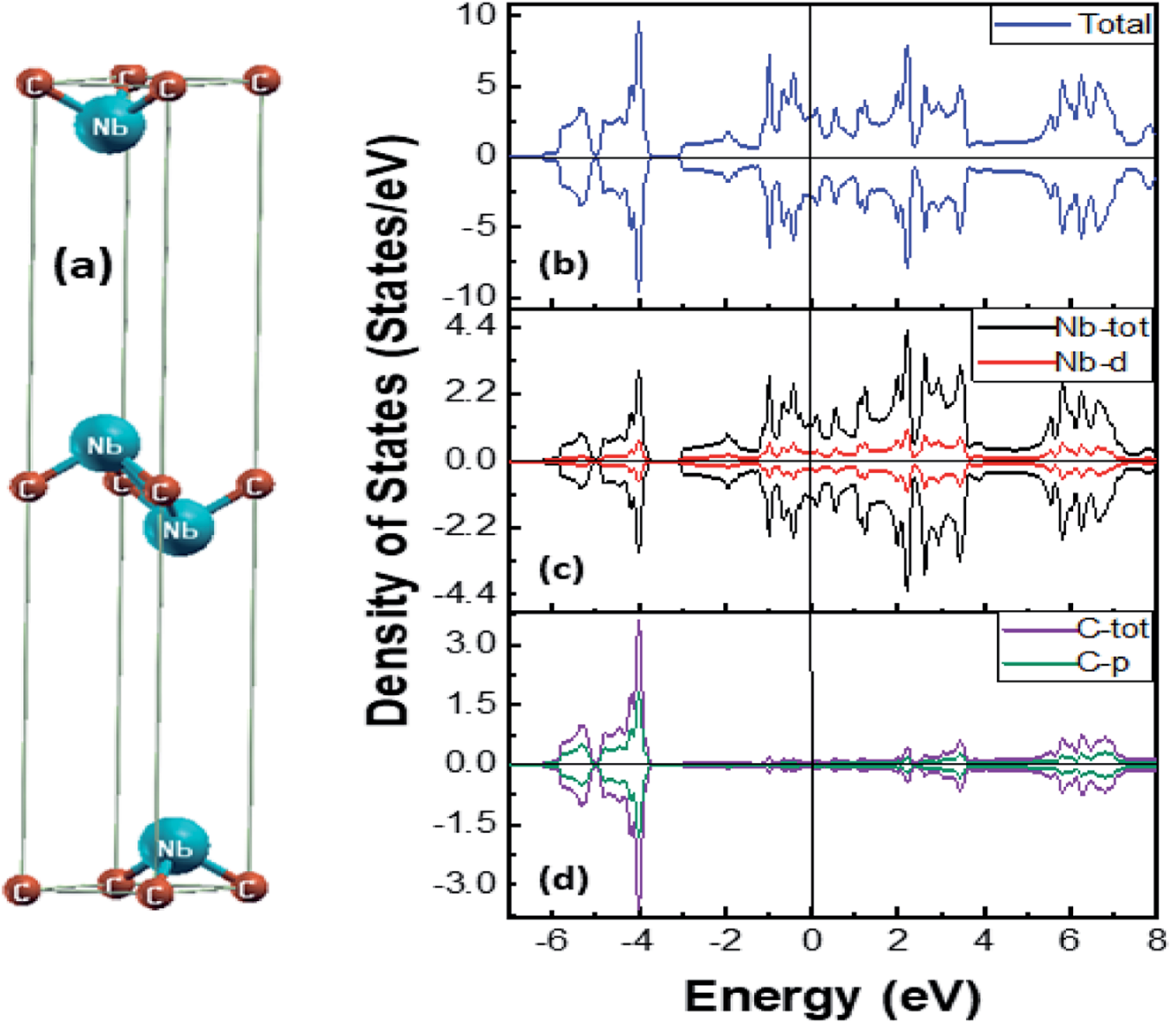
(a) Crystal structure of Nb_2_C in unit cell with space group *P*6_3_/*mmc*. (b) Total density of states for Nb_2_C, (c) total DOS of Nb with partial DOS of Nb-3d shell, and (d) total DOS of C and partial DOS of C-2p shell.

The crystal structure of Nb_2_C is shown in Fig. 6a. The magnetic moment of the compound is calculated to be −0.00485 μB, which although small is yet important, as the value is negative, which is an indication of the presence of diamagnetism in Nb_2_C. Moreover, the magnetic moment of Nb and C, as well as the interstitial position, are −0.00046 μB, −0.00017 μB, and −0.00485 μB, respectively. From the density of states (DOS) calculations, DOS *versus* energy graph is obtained, where Fig. 6b–d shows the total DOS for the compound, total DOS for Nb and partial DOS (PDOS) for the d-orbital of Nb, and total DOS of C with PDOS of the p-orbital of C. In Fig. 6b, at Fermi energy, there is a very small difference between the DOS of spin up and spin down.

Fig. 6c and d clarifies the reason for the negative moment, which is the d-orbital of Nb_2_C, whereas for C, the majority of states present is the valence band, and for Nb, the DOS is spread through the energy range, *i.e.*, valence band and conduction band. Lei *et al.* have studied the different phases of Mo_2_C with functional groups and suggested superconducting behavior. The critical temperature varied according to the functional groups and was found to be in the range of 0 K to 13 K.^76^ Baber *et al.* showed that pristine MXene Nb_2_C exhibits type-II superconductor-like behavior. Magnetization *versus* applied field curves showed that the behavior is diamagnetic below 12.5 K.^8^ The negative total magnetic moment, *i.e.*, −0.00485 μB in the present calculation, is an indication towards that diamagnetic behavior of Nb_2_C. The abovementioned computational studies verify the experimental superconductor-like diamagnetic nature of Nb_2_C MXene.

## Experimental section

The materials used for the etching process include hydrofluoric acid (50 wt% in H_2_O, ≥99.99%), niobium aluminum carbide (Nb_2_AlC) powder (95% pure), de-ionized water, and absolute ethanol.

### Synthesis of Nb_2_C MXene

To obtain Nb_2_C MXene, the wet chemical etching route was followed. The Nb_2_AlC MAX powder (200 mesh size) was immersed in 48–50% concentrated HF (1 g : 10 ml, Sigma Aldrich) at room temperature. Etching at continuous magnetic stirring for 90 hours^4^ was not successful. We started etching at an optimized time of 40 hours while varying the temperature from 45 °C to 85 °C in equal intervals of 10 °C. After the etching process was completed, centrifugation was done, and each sample was washed repeatedly with deionized water. After removing the supernatant, the resulting powder was collected using ethanol and was left at room temperature to dry. The synthesis schematics and detailed etching mechanism are shown in Fig. 1. The rudimental yield comes out to be approximately 100% (yield here is defined as the ratio of the mass obtained for MXene after etching with the mass of MAX taken at the start). The best sample was the one etched at 55 °C, for an optimized etching time of 40 hours. This etching scheme came out to be effective to obtain the required accordion-like layered MXene structure.

### HF handling protocol

It is always risky to handle hydrofluoric acid owing to its harmful and noxious attributes irrespective of its concentration. HF effectively penetrates into the skin, causing damage to deep layer tissues and bones without any visible effects within 1–24 hours.^77^ This leads to the death of tissues and limb loss. So, it is directed to use safety goggles, acid-resistant apron, face-shield and polyvinyl chloride (PVC) or polythene gloves to avoid any direct contact of HF with your skin. Since the inhalation of HF can cause swelling of the respiratory tract, in order to avoid it, the reactions should be carried out in a protective fume hood along with the usage of masks at all times.^78,79^

## Conclusion

In conclusion, we have successfully synthesized 2D-Nb_2_C MXene sheets *via* wet-chemical etching using hydrofluoric (HF) acid. Structural analysis indicated the effective removal of the Al layer, and SEM images show the formation of the resultant layered structure. FTIR spectra indicate the presence of surface terminations that are unavoidable during the etching process. The optical analysis shows a reduction in bandgap that was characterized due to the presence of surface functionalization. Raman spectra show that the MXene is more ordered than the MAX itself. The magnetic analysis shows the variation of the magnetic behavior of MXene upon application of different field strengths as well as entirely different behaviors of MAX and MXene. Our theoretical analysis validates the diamagnetic nature of MXene. Although the value of the negative magnetic moment is small, it very importantly signifies the experimental diamagnetic nature of MXene, giving clear signatures of the possible existence of superconductivity in Nb_2_C MXene, which can be verified further by transport measurement to make it a 100% certainty in the future. It is notable that to our knowledge, this is the first study on Nb_2_C-MXene that conducts experimental studies in combination with the theoretical validation of experimental findings. For our MXene, these physical properties are significant, which could make it a leading member of the 2D MXene family in terms of its novel magnetic nature for possible application in two-dimensional spintronics.

## Conflicts of interest

The authors declare no competing financial interest.

## Supplementary Material
